# The complete mitochondrial genome of *Calliptamus barbarus* Costa 1836 (Orthoptera: Acrididae) from Qinghai Lake, China and its phylogeny

**DOI:** 10.1080/23802359.2020.1860701

**Published:** 2021-03-11

**Authors:** Jun Wang, Lei Tang

**Affiliations:** a State Key Laboratory of Hydrology-Water Resources and Hydraulic Engineering, Nanjing Hydraulic Research Institute, Nanjing, China; b Center for Eco-Environmental Research, Nanjing Hydraulic Research Institute, Nanjing, China

**Keywords:** *Calliptamus barbarus*, Orthoptera, Acrididae, mitogenome, phylogenetic analysis

## Abstract

The complete mitochondrial genome of *Calliptamus barbarus* (Orthoptera: Acrididae: Calliptaminae) from Qinghai Lake, Qinghai province, China is a circular molecule of 15,668 bp in size, and contains 13 protein-coding genes, 22 transfer RNA genes, 2 ribosomal RNA genes, and one AT-rich region. The overall nucleotide composition is 41.8% of A, 30.9% of T, 11.3% of G, and 16.0% of C. All PCGs started with typical ATN codon, e.g. one with ATA, two with ATT and ATC, and eight with ATG. Eleven PCGs ended with complete stop codon TAA, and the other two genes (ND1 and ND4L) ended with TAG. Phylogenetic trees were reconstructed with 13 PCGs using Bayesian Inference (BI) and maximum likelihood (ML) to validate the taxonomic status of *C. barbarus*, exhibiting the close relationships with *C. abbreviates + C. italicus*.

The complete mitochondrial genome (mitogenome) of insect is usually a circular molecule spanning 14–20 kb in length (Boore 1999). Owing to several properties of rapid evolutionary rate, small genome size, low recombination, and maternal inheritance, mitogenomes are being increasingly employed to explore the evolution and phylogenetic relationships in diverse insect taxa (Cameron 2014; Dai et al. 2018). *Calliptamus barbarus* Costa 1836 is one species of the genus *Calliptamus*, belongs to the subfamily Calliptaminae, within the family Acrididae of the order Orthoptera (Cigliano et al. 2020). The barbarine grasshopper is an insect considered as a potential pest of crops, especially during outbreaks (Rouibah et al. 2018). In this study, we determined and characterized the complete mitochondrial genome of *C. barbarus*, contributing to aid further phylogenetic and genetic studies of this polymorphic species.

Adult specimens of *C. barbarus* were collected in Qinghai Lake (37°43′N, 100°48′E), Qinghai province, China. After morphological identification, the collected specimens were stored in 100% ethanol and kept in the laboratory of Nanjing Hydraulic Research Institute, Nanjing, Jiangsu (the voucher No. WJ2020CB). Total genomic DNA was extracted from hind femoral muscles of each specimen using a Wizard^®^ Genomic DNA Purification Kit (Promega, Madison, USA) according to the manufacturer’s instructions. The mitogenome of *Calliptamus italicus* (GenBank accession No. EU938373) was employed as the reference sequence. Certain pairs of universal primers for loucst mitochondrial genomes were used for polymerase chain reaction (PCR) amplification (Simon et al. 2006). Then PCR products were sequenced using primer-walking strategy from both strands by Genscript Biotech Corp. (Nanjing, China). The mitochondrial genome was assembled by SeqMan program from DNASTAR (Burland 2000) and annotated using MITOS Web Server (Bernt et al. 2013).

The complete mitogenome of *C. barbarus* (Genbank accession No. MT985324) was sequenced to be 15,668 bp in size. The mitogenome consisted of 13 typical protein-coding genes (PCGs), 22 transfer RNA genes (tRNAs), two ribosomal RNA genes (rRNAs), and one AT-rich region, which is similar with the typical mitogenome of other orthopterans (Li et al. 2020). Like other acridid mitogenomes, 24 genes were encoded on the H-strand and the other 13 lay on the L-strand. The overall nucleotide composition was 41.8% of A, 30.9% of T, 11.3% of G, and 16.0% of C. All PCGs started with typical ATN codon, e.g. one with ATA, two with ATT and ATC, and eight with ATG. Eleven PCGs ended with complete stop codon TAA, and the other two genes (ND1 and ND4L) ended with TAG. The 12S (846 bp) and 16S (1377 bp), were located between the tRNA^Leu1^ and AT-rich region, and separated by the tRNA^Val^ gene. 22 tRNA genes range in size from 64 to 71 bp. All tRNAs harbored the typical predicted secondary cloverleaf structures except for the tRNA^Ser1^, as seen in all other determined locusts (Li et al. 2020).

To validate the phylogenetic position of *C. barbarous* in Acrididae, the Maximum Likelihood (ML) and Bayesian Inference (BI) trees were constructed on CIPRES Portal using 13 PCGs from mitogenomes of 22 acridid species and two outgroups, respectively (Figure 1). We used the best-fit partitioning scheme and partition-specific models recommended by PartitionFinder (Lanfear et al. 2012). Two phylogenetic analyses using different methods yielded the same topology, and nodal supporting values were always higher for BI tree than for ML tree (Figure 1). As shown in Figure 1, *C**. barbarus* was the sister clade with *C. abbreviates* + *C. italicus* within the genus of *Calliptamus*, which indicated that our newly determined mitogenome sequence could meet the demands and explain some evolution issues.

**Figure 1.**
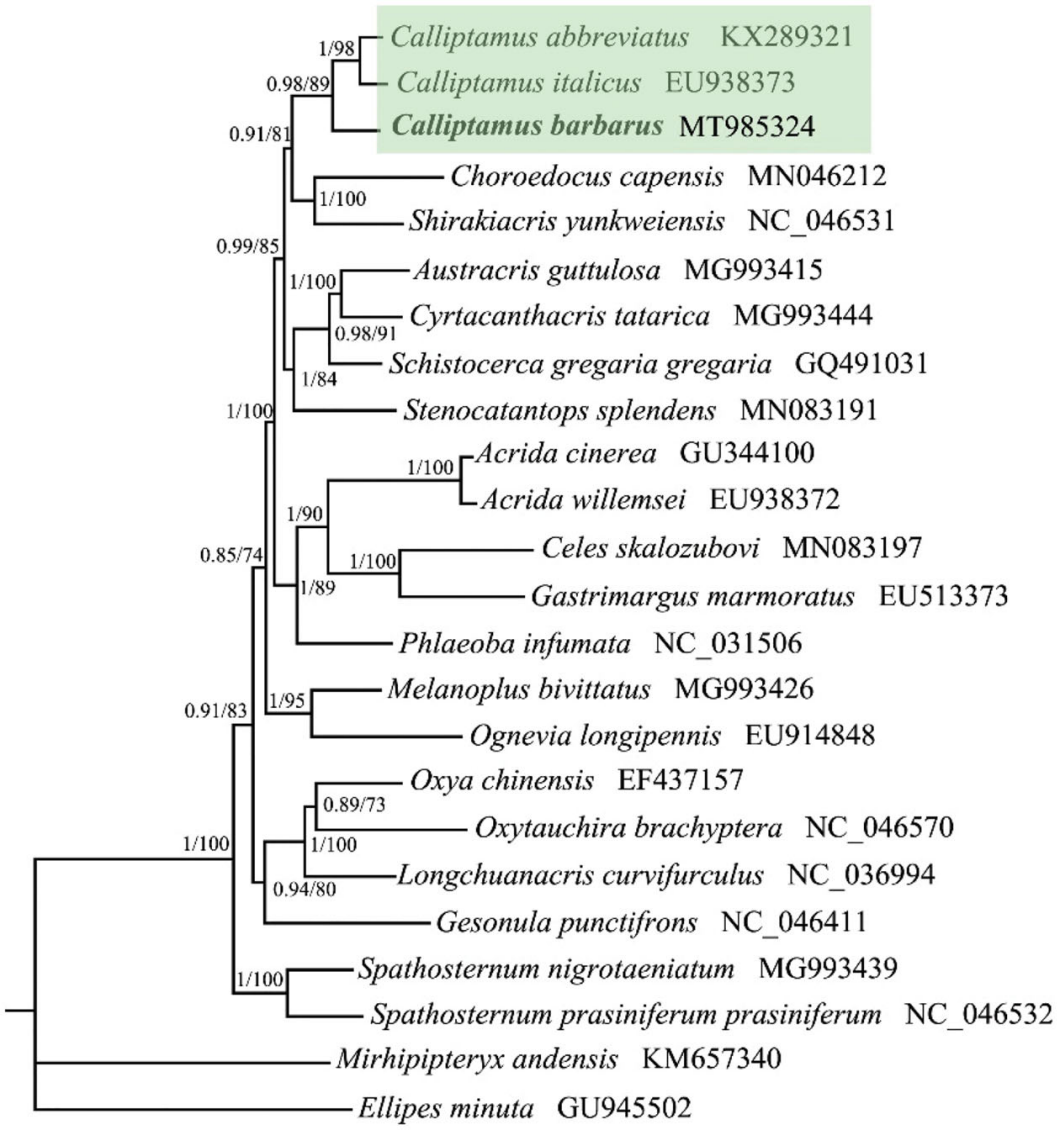
Phylogenetic tree obtained from ML and BI analysis based on 13 concatenated mitochondrial PCGs. Numbers separated by a slash on node are posterior probability (PP) and bootstrap value (BV).

## Data Availability

The genome sequence data that support the findings of this study are openly available in GenBank of NCBI at [https://www.ncbi.nlm.nih.gov](https://www.ncbi.nlm.nih.gov/) under the accession no. MT985324.
